# Resurrection and redescription of *Varestrongylus alces* (Nematoda: Protostrongylidae), a lungworm of the Eurasian moose (*Alces alces*), with report on associated pathology

**DOI:** 10.1186/s13071-014-0557-8

**Published:** 2014-12-17

**Authors:** Guilherme G Verocai, Eric P Hoberg, Turid Vikøren, Kjell Handeland, Bjørnar Ytrehus, Andrew M Rezansoff, Rebecca K Davidson, John S Gilleard, Susan J Kutz

**Affiliations:** Department of Ecosystem and Public Health, Faculty of Veterinary Medicine, University of Calgary, 3280 Hospital Drive NW, Calgary, Alberta T2N 4Z6 Canada; US Department of Agriculture, United States National Parasite Collection, Agricultural Research Service, BARC East No. 1180, 10300 Baltimore Avenue, Beltsville, Maryland 20705 USA; Norwegian Veterinary Institute, Ullevålsveien 68, N-0454 Oslo, Norway; Present address: Norwegian Institute for Nature Research, P.O. box 5685, Sluppen N-7485 Trondheim, Norway; Department of Comparative Biology and Experimental Medicine, Faculty of Veterinary Medicine, University of Calgary, 3330 Hospital Drive NW, Calgary, Alberta T2N 4N1 Canada; Present address: Norwegian Defence Research Institute, Postboks 25, 2027 Kjeller, Norway; Canadian Wildlife Health Cooperative - Alberta Node, Faculty of Veterinary Medicine, University of Calgary, 3280 Hospital Drive NW, Calgary, Alberta T2N 4Z6 Canada

**Keywords:** Cervidae, Cryptic species, Historical biogeography, ITS-2, Metastrongyloidea, Parasite biodiversity, Varestrongylinae, *Varestrongylus capreoli*, Verminous pneumonia

## Abstract

**Background:**

*Varestrongylus alces*, a lungworm in Eurasian moose from Europe has been considered a junior synonym of *Varestrongylus capreoli*, in European roe deer, due to a poorly detailed morphological description and the absence of a type-series.

**Methods:**

Specimens used in the redescription were collected from lesions in the lungs of Eurasian moose, from Vestby, Norway. Specimens were described based on comparative morphology and integrated approaches. Molecular identification was based on PCR, cloning and sequencing of the ITS-2 region of the nuclear ribosomal DNA. Phylogenetic analysis compared *V. alces* ITS-2 sequences to these of other *Varestrongylus* species and other protostrongylids.

**Results:**

*Varestrongylus alces* is resurrected for protostrongylid nematodes of Eurasian moose from Europe. *Varestrongylus alces* causes firm nodular lesions that are clearly differentiated from the adjacent lung tissue. Histologically, lesions are restricted to the parenchyma with adult, egg and larval parasites surrounded by multinucleated giant cells, macrophages, eosinophilic granulocytes, lymphocytes. The species is valid and distinct from others referred to *Varestrongylus*, and should be separated from *V. capreoli*. Morphologically, *V. alces* can be distinguished from other species by characters in the males that include a distally bifurcated gubernaculum, arched denticulate crura, spicules that are equal in length and relatively short, and a dorsal ray that is elongate and bifurcated. Females have a well-developed provagina, and are very similar to those of *V. capreoli*. Morphometrics of first-stage larvae largely overlap with those of other *Varestrongylus*. Sequences of the ITS-2 region strongly support mutual independence of *V. alces*, *V.* cf. *capreoli*, and the yet undescribed species of *Varestrongylus* from North American ungulates. These three taxa form a well-supported crown-clade as the putative sister of *V. alpenae*. The association of *V. alces* and *Alces* or its ancestors is discussed in light of host and parasite phylogeny and host historical biogeography.

**Conclusions:**

*Varestrongylus alces* is a valid species, and should be considered distinct from *V. capreoli*. Phylogenetic relationships among *Varestrongylus* spp. from Eurasia and North America are complex and consistent with faunal assembly involving recurrent events of geographic expansion, host switching and subsequent speciation.

## Background

The Family Protostrongylidae Leiper, 1926 (Metastrongylina) is comprised of six subfamilies: Protostrongylinae Kamensky, 1905; Muelleriinae Skrjabin, 1933; Elaphostrongylinae Boev & Shulz, 1950; Neostrongylinae Boev & Shulz, 1950; Skrjabincaulinae Boev & Sulimov, 1963; and Varestrongylinae Boev, 1968 [1,2]. Representative species of all subfamilies occur in the Palaearctic, and are often pathogenic parasites of Artiodactyla, especially cervids and caprines, and Lagomorpha. Adult nematodes of species within Varestrongylinae, including those within the genus *Varestrongylus* Bhalerao, 1932, reside in the lung parenchyma, bronchi and bronchioles of their hosts, and cause verminous pneumonia [3-5]. Similar to other protostrongylids, definitive hosts are infected by *Varestrongylus* spp. through ingestion of infective third-stage larvae (L3) contained within gastropod intermediate hosts (IH) or, possibly, L3 that have emerged from the gastropods [6-8].

The majority of species within *Varestrongylus* are endemic to Eurasia, which is the centre of diversity for this genus and their hosts [2,9-11]. Currently, the Eurasian biodiversity of *Varestrongylus* includes seven species, infecting an array of hosts within Bovidae (Caprinae) and Cervidae (Cervinae and Odocoileinae or Capreolinae *sensu* [12]): *Varestrongylus sagittatus* (Mueller 1890), *Varestrongylus pneumonicus* Bhalerao, 1932, *Varestrongylus capreoli* (Stroh & Schmidt [3]), *Varestrongylus capricola* Sarwar, 1944, *Varestrongylus tuvae* (Boev & Sulimov, 1963), *Varestrongylus qinghaiensis* Liu, 1984 and *Varestrongylus longispiculatus* Liu, 1989 [1,6,13,14]. This Eurasian fauna is significantly richer when contrasted with the diversity *Varestrongylus* in the Nearctic which, to date, includes only one described species, *Varestrongylus alpenae* (Dikmans 1935), and an as yet undescribed taxon that is known from sequence data and first stage larvae [15-17].

Not surprisingly, given its diverse nature, the taxonomic history for this genus has been markedly unstable, with several taxa having inconsistently been reduced as junior synonyms [1,18-20]. One such example is *V. alces*, originally described in the Eurasian moose (also known as Eurasian elk) (*Alces alces* L.) from Russia [21]. *Varestrongylus alces* was later synonymized with *V. capreoli* Stroh & Schmidt [3] in European roe deer (*Capreolus capreolus* (L.) [1]. Synonymy was due primarily to a vague, poorly illustrated description and assumptions about host distributions for these parasites, confounded by the absence of a designated type series deposited in a museum collection [1,21]; Arseny Makarikov, pers. comm.].

Despite apparent taxonomic confusion around the validity of *V. alces*, many authors continued to report this varestrongyline, usually as an incidental finding under various names including *V. capreoli*, *V. alces*, *Bicaulus alces or* ‘*Bicaulus alcis’* (sic). These identifications do not appear to have been confirmed through careful morphological examination, nor were these survey collections accompanied by voucher specimens in a recognized repository [22-28]. An additional factor that might have drawn attention away from *V. alces* was the description of the pathogenic, *Elaphostrongylus alces* Stéen, Chabaud & Rehbinder [29]. This meningeal nematode, which shares its host and geographic range with *V. alces*, has irrefutable veterinary importance, causing neurologic disease in affected hosts, and commonly occurs in co-infections with its less pathogenic, pulmonary relative *V. alces* [29,30]; additionally both species have dorsal-spined first stage larvae that would be largely indistinguishable.

Herein, using combined morphological and molecular approaches, we resurrect and redescribe *V. alces*, a protostrongylid lungworm in Eurasian moose. A proposal for designation of a neotype specimen and an associated series is presented. We report associated gross and histopathological findings, and comment on phylogenetic relationships among selected *Varestrongylus* species, their host-associations and biogeography.

## Methods

### Specimen collection

Lungs of 13 Eurasian moose were examined for the presence of lungworms at the wildlife unit of the Norwegian Veterinary Institute (NVI), Oslo between October and December, 2011. All animals were harvested in the municipality of Vestby (59°30′N, 10°40′E), County of Akershus, East Norway Region, Norway.

Additional varestrongyline specimens, attributable to *V. capreoli* (hereafter named *V.* cf*. capreoli*) were recovered from lungs of two European roe deer at the NVI, an adult male and a female calf, from the same region.

Lungs from Eurasian moose and roe deer were examined for lungworms. Gross lesions consistent with *Varestrongylus* infection were removed, placed in saline solution, and finely dissected to isolate adult nematodes. All intact worms or fragments of anterior and posterior extremities were collected, identified by gender, and stored in tagged vials containing 70% ethanol. The lung samples were also flushed with saline in order to isolate larvae and eggs. These were fixed in steaming 70% ethanol.

### Morphological identification

Adult specimens and fragments containing relevant morphological characters were mounted and cleared in phenol-alcohol, and examined under differential interference contrast microscopy (Table 1). In the redescription, measurements are in micrometers unless specified otherwise, and are presented with the numbers of adult male, female and larval nematodes examined (n =), and the range is followed by the mean ± 1 SD in parentheses. Adult specimens of other species of *Varestrongylus* were mounted and cleared in phenol-alcohol and examined microscopically. These included some species in potential sympatry with *V. alces*, and other prominent taxa in cervids (Table 2).

**Table 1:** **Lungworm material collected and/or used in the study**

USNPC*	***Varestrongylus*** **species**	**Host**	**Country**	**Specimens**	GenBank**
106331	*V. alces* Demidova & Naumitscheva 1953	*Alces alces* ^a^	Norway	1♂	KJ452181-83
106332	*V. alces*	*A. alces* ^a^	Norway	1♂	KJ452188-90
106333	*V. alces*	*A. alces* ^a^	Norway	3♀	NA
106334	*V. alces*	*A. alces* ^a^	Norway	DSL	NA
106335	*V. alces*	*A. alces* ^b^	Norway	1♂, 2♀	NA
106336	*V. alces*	*A. alces* ^c^	Norway	2♂, 3♀	NA
106337	*V. alces*	*A. alces* ^d^	Norway	1♂ (neotype)	NA
106338	*V. alces*	*A. alces* ^d^	Norway	2♂, 3♀	NA
106339	*V. alces*	*A. alces* ^d^	Norway	♀	KJ452195-96
106340	*V. alces*	*A. alces* ^d^	Norway	♂^g^	KJ452191-94
NA	*V. alces*	*A. alces* ^d^	Norway	fragment	KJ452184-87
106341	*V.* cf. *capreoli*	*Capreolus capreolus* ^e^	Norway	6♂,5♀	NA
106342	*V.* cf. *capreoli*	*C. capreolus* ^e^	Norway	1♀	KJ452177-80
106343	*V.* cf. *capreoli*	*C. capreolus* ^e^	Norway	1♀	NA
106344	*V.* cf. *capreoli*	*C. capreolus* ^f^	Norway	1♀, DSL	NA
NA	*V.* cf. *capreoli*	*C. capreolus* ^e^	Norway	fragment	KJ452174-76
104105	*V. sagittatus* (Mueller 1890)	*Cervus elaphus*	Bulgaria	1♂	KJ439592-95
104105	*V. sagittatus*	*C. elaphus*	Bulgaria	1♀	KJ439596-99

*Museum accession numbers; Additional host information (Eurasian moose): a. V-376, yearling female; b. V-377, yearling female; c. V-383, adult female; d. V-456, yearling male. Roe deer - e. V-379, adult male; f. V-510, adult female; g. broken specimen, not used for morphometry. **Number of ITS-2 sequences varies according to number of clones yielded from DNA lysates of each individual worm.

Lungworm material collected and/or used in the study, with information on host and origin, and matching accession numbers for specimens deposited at the United States National Parasite Collection (USNPC) and sequences at the internal transcribed spacer-2 locus of the nuclear ribosomal DNA (ITS-2) deposited in GenBank.

**Table 2:** **Additional** ***Varestrongylus*** **specimens from the United States National Parasite Collection (USNPC) morphologically examined**

USNPC*	***Varestrongylus*** **species**	**Host**	**Locality**	**Specimens**
34066	*V. alpenae* (Dikmans 1935)	*Odocoileus virginianus*	Michigan, USA	1♂ (holotype)
78599	*V. alpenae*	*O. virginianus*	Alberta, Canada	2♂, 1♀
37833	*V. pneumonicus* Bhalerao, 1932^a^	*Ovis aries*	Alma-Ata, Kazakhstan	1♂
37834	*V. pneumonicus* ^a^	*O. aries*	Alma-Ata, Kazakhstan	1♂
45106	*V. pneumonicus* ^b^	*O. aries*	Lanchow, China	2♂, 2♀
37851	*V. sagittatus* (Mueller 1890)^c^	*Cervus elaphus*	Altai Mtns., Kazakhstan	1♀
37855	*V. sagittatus*	*C. elaphus*	Altai Mtns., Kazakhstan	1♂
89171	*V. sagittatus*	*C. elaphus*	Altai Region, Russia	1♂, 1♀

*Museum accession numbers; ^a^referred as *Bicaulus schulzi* (Boev and Wolf 1938); ^b^referred as *V. sinicus* Dikmans 1945; ^c^referred as *Bicaulus sagittatus* (Mueller 1890).

Eggs and first-stage dorsal-spined larvae (DSL) recovered from the lungs of one Eurasian moose (V-376) were microscopically examined. Measurements are in micrometers.

Specimens of *V*. cf. *capreoli* and *V. sagittatus* (Table 1), collected respectively from the lungs of European roe deer from Norway (by the authors) and the European red deer (*Cervus elaphus*) in Bulgaria (by M. S. Panayotova-Pencheva), were processed for molecular-based comparisons according to methodology described below; sequences produced for both species were included in the phylogenetic analysis.

### Gross and histopathology

Gross pathologic changes in Eurasian moose lungs were documented during necropsy and dissection. Sections of fresh lung tissue were collected from one Eurasian moose (V-456), fixed in 10% neutral buffered formalin, embedded in paraffin, sectioned at 5 μm and stained with haematoxylin and eosin (H&E) and van Gieson (VG) for histological examination.

### Molecular analyses

#### DNA extraction and amplification

Genomic DNA (gDNA) was extracted from small fragments of adult nematodes in 0.2 mL tubes containing 5 μL of deionized water and 25 μL of lysis buffer (0.5 mg/mL of proteinase K, 10× PCR buffer). The following DNA extraction protocol was used: tubes containing adult worm fragments were incubated at 60°C for 60 min, 65°C for 60 min, then at 95°C for 15 min. Extracted DNA was diluted 1:10. For species identification, a PCR was performed using primers NC1 (5′-ACG TCT GGT TCA GGG TTG TT-302B9) and NC2 (5′-TTA GTT TCT TTT CCT CCG CT-3′) targeting the ITS-2 region of the nuclear ribosomal DNA [15,31]. PCR amplification was performed in 40 μL reactions containing: 20.4 μL of water, 8 μL of 10× PCR buffer + MgCl_2_, 0.8 μL of 10 mmol dNTPs, 4 μL (10 μM) of each primer, 0.4 μL of bovine serum albumin,0.4 μL of *Taq* Phusion HF DNA polymerase, and 2 μL of DNA template. The amplification conditions used were an initial 2 min denaturation at 98°C, followed by 35 cycles of 98°C for 10 s, 52.5°C for 30 s, and 72°C for 30 s. A final extension phase of 72°C for 5 min was followed by cooling to 10°C [31].

#### Cloning and sequencing

PCR products were gel-purified using e.Z.N.A MicroElute® Gel Extraction Kit (Omega Biotek) following the manufacturer’s protocol. All 40 μL of the reactions were used. Gel-purified DNA was eluted in 15 μL nuclease free water. Gel purified DNA amplicons were then ligated using CloneJET PCR Cloning Kit according to manufacturer’s instructions and transformed into Subcloning Efficiency™ DH5α™ Competent Cells. After overnight incubation on standard LB agar bacterial plates with 100 μg/mL ampicillin, four colonies were randomly selected from plates of each individual, and re-colonized in 3 mL LB broth. After a second overnight incubation these cultures were centrifuged to attain bacterial pellets, for which and plasmid DNA was prepared using e.Z.N.A Plasmid Mini-Kit I (Omega Biotek). Plasmid DNA isolates were then sequenced using NC1 and NC2 primers on BigDye Terminator Cycle Sequencing platform (Applied Biosystems).

### Sequence analysis

A total of 31 clonal sequences representing 9 individuals (16 clones from 5 *V. alces* specimens, 7 clones from 2 *V.* cf. *capreoli*, 8 clones from 2 *V. sagittatus* individuals) passed quality control and were included in the analysis using Geneious Pro [32]. Once fully processed the 31 clones were realigned to attain pairwise distances among clones and other protostrongylid ITS-2 sequences available in GenBank.

### Phylogenetic analysis

**Figure 1:**
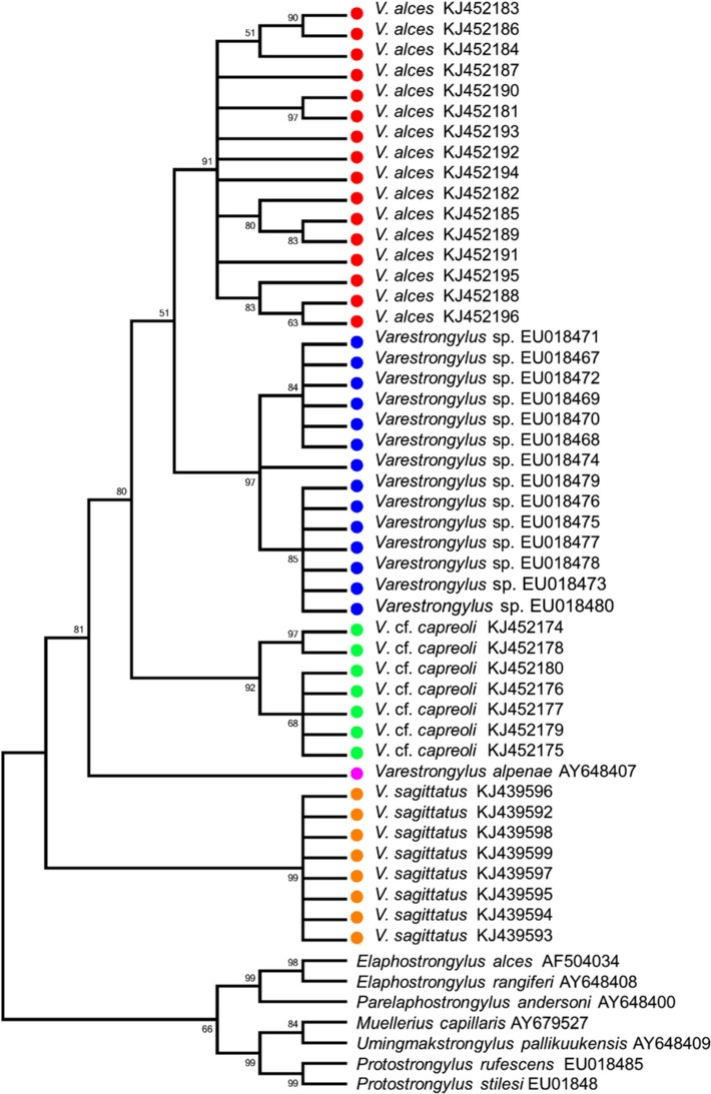
**Phylogenetic relationships among** ***Varestrongylus*** **species and other Protostrongylidae.** Most-parsimonious tree depicting the independence of *Varestrongylus alces* and other *Varestrongylus* species, and the reciprocal monophyly of sequences within each. The bootstrap consensus tree inferred from 5,000 replicates is taken to represent the evolutionary history of the taxa analyzed. Branches corresponding to partitions reproduced in less than 50% bootstrap replicates are collapsed. The percentage of replicate trees in which the associated taxa clustered together in the bootstrap test (5,000 replicates) shown next to the branches [33].

Cloned ITS-2 sequences produced in this study for *V. alces*, *V.* cf. *capreoli* and *V. sagittatus* were compared to those of *V. alpenae*, and an undescribed species of *Varestrongylus* in wild North American ungulates [15]. Broader comparisons involved other protostrongylids examined in prior studies (e.g., [15]) with sequence data obtained from GenBank including representatives of Elaphostrongylinae (*E. alces*, *E. rangiferi* and *P. andersoni*), Muelleriinae (*Muellerius capillaris* (Mueller 1889), *Cystocaulus ocreatus* (Railliet & Henry, 1908), and *Umingmakstrongylus pallikuukensis* Hoberg, Polley, Gunn & Nishi, 1995) and Protostrongylinae (*Protostrongylus rufescens* (Leuckart, 1965) and *Protostrongylus stilesi* Dikmans, 1931) (accession numbers in Figure 1). Sequences were aligned using PRANK, a probabilistic multiple alignment program available through the European Bioinformatics Institute (http://www.ebi.ac.uk/goldman-srv/prank). Aligned sites were not filtered by posterior probability. Phylogenetic reconstruction analysis was performed using the maximum parsimony (MP) method in MEGA 5.2 [33], with gaps treated as complete deletion (100%), sub-tree pruning regrafting as MP search model, and 5,000 bootstrap replicates.

Intra- and interspecific pairwise similarity was calculated for ITS-2 sequences of six different *Varestrongylus* spp., including the sequenced clones, using the distance matrix generated by Geneious Pro [32].

Specimens of *V*. cf. *capreoli* and *V. sagittatus* (Table 1), collected respectively from the lungs of European roe deer from Norway (by the authors) and the European red deer (*Cervus elaphus*) in Bulgaria (by M. S. Panayotova-Pencheva), were processed for molecular-based comparisons according to methodology described below; sequences produced for both species were included in the phylogenetic analysis.

## Results

Nematode specimens used for this redescription of *V. alces* were isolated from the lungs of four (30.8%, n = 13) Eurasian moose. Infected hosts were: an adult female (V-383), two yearling females (V-376, V-377) and a yearling male (V-456).

### Redescription

#### Varestrongylus alces Demidova & Naumitscheva, 1953

Syn.: *Bicaulus alces* (Demidova & Naumitscheva, 1953) Boev, 1957; *Varestrongylus capreoli* (in part., *sensu* Boev, 1975)

### General description

**Figure 2:**
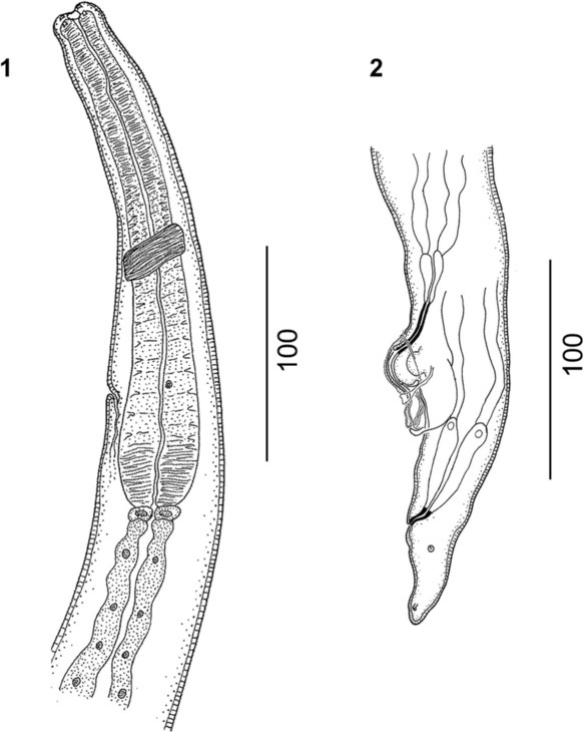
***Varestrongylus alces.*** Female. 1. Cephalic extremity of a female specimen at ventral view. 2. Caudal extremity of a female specimen at lateral view, showing a developed provagina.

**Figure 3:**
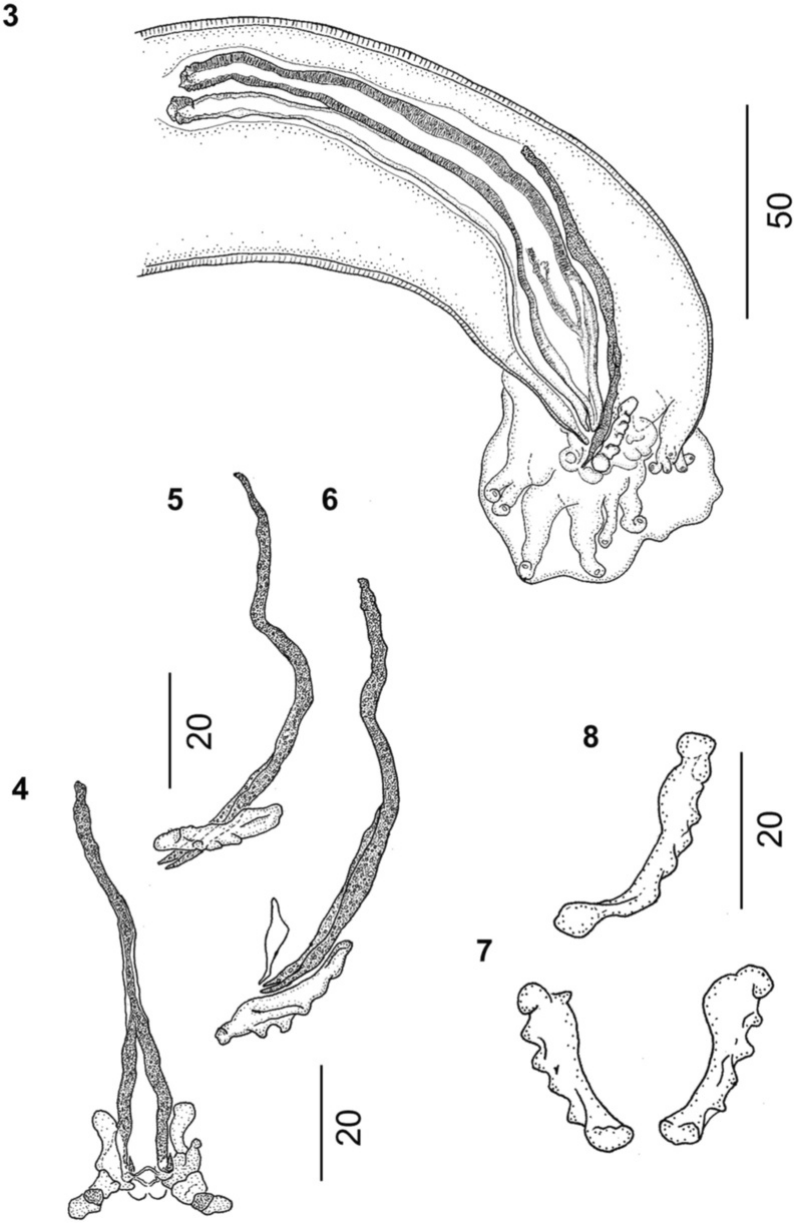
***Varestrongylus alces.*** Male. 3. Caudal extremity of a male specimen at lateral view showing spicule, partially covering gubernaculum and denticulate plates of crura and copulatory bursa; 4. Ventral view of bifurcate gubernaculum; 5, 6. Lateral view of gubernaculum and denticulate plates of crura, note triangular telamon plate in 6. 7. Ventral view of paired denticulate plates of crura. 8. Lateral view of a denticulate plate of crura.

**Figure 4:**
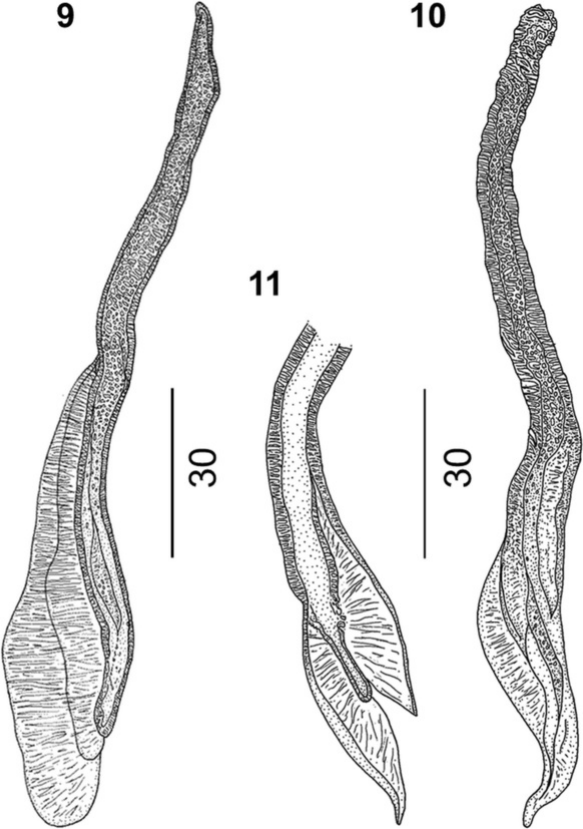
***Varestrongylus alces.*** Male, spicules. 9. Dorsal view, note prominent alae and spatulate shape. 10. Lateral view. 11. Ventral view of spicule distal end.

**Figure 5:**
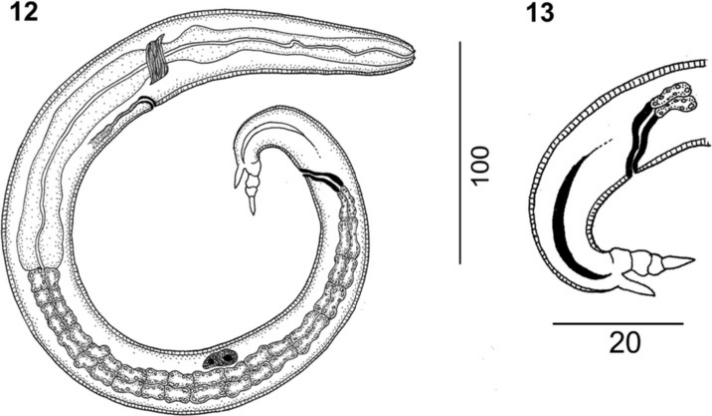
***Varestrongylus alces.*** First-stage larva (DSL). 12. DSL at lateral view. 13. Detail on caudal extremity, note dorsal spine and tail extremity composed by three segments.

**Figure 6:**
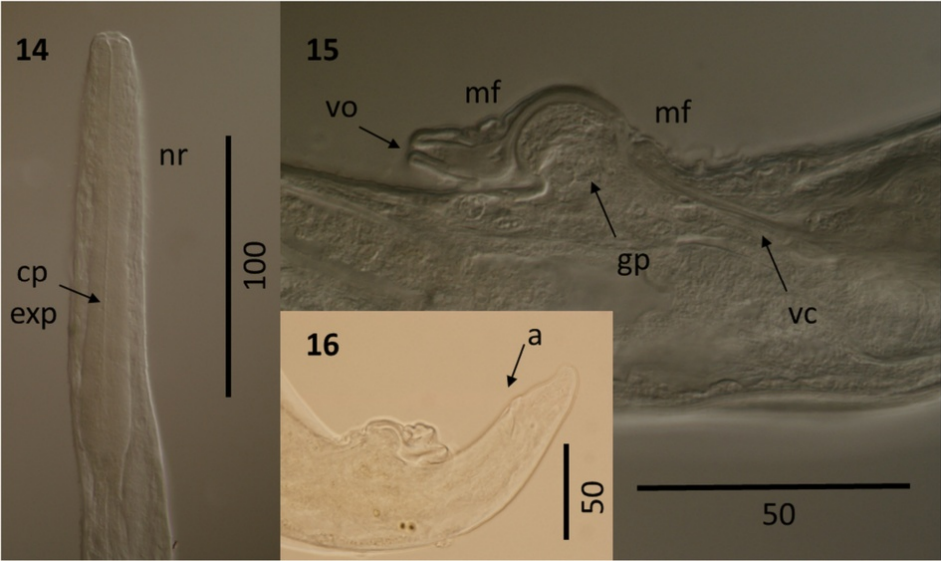
***Varestrongylus alces.*** Female. 14. Cephalic extremity at ventral view: claviform esophagus, cervical papillae (cp), excretory pore (exp), and nerve ring (nr) (64×). 15. Caudal extremity at lateral view: developed provagina with membranous folds (mf), genital protuberance (gp), vaginal opening (vo), and vaginal canal (vc) (100×). 16. Caudal extremity at lateral view, slightly ventral: anus (a), and conical tail tip (100×).

**Figure 7:**
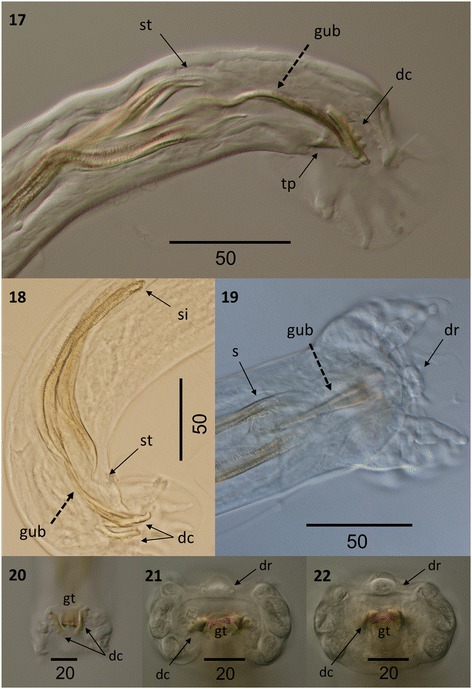
***Varestrongylus alces.*** Male. 17. Caudal extremity of a male specimen at dorsal view: arched bifurcate gubernaculum (gub), spatulate spicule tips (st), denticulate plates of crura (dc) and triangular telamon plate (tp) (64×). 18. Caudal extremity of a male specimen at lateral view: spicule insertion (si) and spatulate tips (st), bifurcate gubernaculum (gub), and paired denticulate plates of crura (dc) (100×). 19. Caudal extremity of a male specimen at ventral view: distal end of spicules (s), bifurcate gubernaculum (gub), and dorsal ray (dr) (40×). 20. Caudal extremity of a male specimen at ventral view: denticulate plates of crura (dc), and tip of gubernaculum (gt) (64×). 21, 22. Detail of male caudal extremity at caudal view: dorsal ray (dr), denticulate crura (dc), and tip of gubernaculum fused by delicate membrane (tg) (160×).

**Figure 8:**
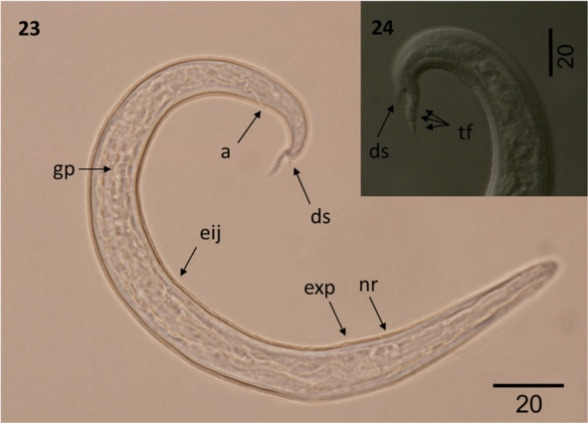
***Varestrongylus alces.*** First-stage larva (DSL). 23. DSL at lateral view (100×): nerve ring (nr), excretory pore (exp), esophageal-intestinal junction (eij), genital primordium (gp), anus (a) and dorsal spine (ds). 24. Detail of tail, showing dorsal spine (ds) and the three tail folds (tf) (100×).

(Figures 1, 2, 3, 4, 5, 6, 7 and 8) Protostrongylidae, Varestrongylinae, thin and minute nematodes, reddish brown prior to fixation with delicate, transversally striated cuticle. Cephalic extremity bluntly rounded. Buccal opening surrounded by four papillae. Esophagus cylindrical, clavate, broader at base, and poorly demarcated in muscular and glandular sections. Nerve ring indistinct, located at anterior or middle third of esophagus. Diminutive cervical papillae and excretory pore located at middle or posterior third of esophagus, always posterior to nerve ring.

### Males

Based on specimens in four Eurasian moose: six intact males, including neotype, and three fragments containing caudal extremities. Total length (n = 6) 11.36–14.7 mm (12.97 ± 2.01); maximum width (n = 6) 68.5–80 (74.2 ± 4.97). Esophagus (n = 5) 250–272 (264.5 ± 8.95) long, 32–37 (33.9 ± 2.19) wide, (n = 5) 1.6–2.3% (2.0 ± 0.28%) of body length. Body width at esophagus (n = 5) 53.8–61.9 (33.9 ± 2.19). Nerve-ring (n = 5) 68–89.65 (81.8 ± 9.04), cervical papillae (n = 3) 201–207 (203.4 ± 3.19), and excretory pore (n = 3) 208–230.3 (221.8 ± 9.83) from cephalic extremity. Copulatory bursa rounded, with indistinct dorsal lobe. Bursal rays approaching, rarely attaining margin of bursa. Body width at bursa (n = 7) 42–56 (48.3 ± 7.36), bursa length (n = 6) 75–90 (84.3 ± 5.86), bursa width (n = 3) 125–160 (140 ± 18.03). Ventro-ventral and latero-ventral rays equal, parallel, arising from common stalk, directed anteriad and isolated, tips of rays distally separate. Lateral rays arising from common base; externo-lateral elongate, attaining bursal margin, isolated from medio- and postero-lateral rays. Externo-lateral and medio-lateral rays of equal length. Medio-lateral rays long, postero-lateral rays reduced, with tips separate from near to less than half of common stalk. Externo-dorsal rays long, origins independent from base of dorsal ray. Dorsal ray elongate (n = 7) 18–30 (24.5 ± 3.65) long, (n = 8) 11.41–15 (12.9 ± 1.51) wide at base. Dorsal ray bifurcate near middle third (n = 5) 12–17 (14.2 ± 1.79) from base, (n = 4) 40–58.3% (51.5 ± 7.95%) of its length. Spicules tubular, equal, symmetrical, yellowish brown, (n = 8) 138.55–163 (153.3 ± 7.31) long, anterior portion short, strongly chitinized, without distal split; prominent bilateral alae with prominent ridges and trabeculae, originating in first third of spicule length from anterior extremity. Alae spatulate, prominent, extending to distal termination of spicule tips. Gubernaculum lacking capitulum, thin, arched, elongate, (n = 8) 65–83.13 (76.6 ± 7.06) composed of single corpus and paired crurae. Unpaired anterior corpus (n = 8) 38–49 (44 ± 4.34), bifurcate distally into two lateral legs near mid-third (n = 8) 24–39.12 (32.6 ± 5.09); distal tips prominent, arched ventrally, joined by delicate membrane, located between, slightly ventral to paired denticulate plates of crurae. Denticulate plates of crurae (n = 8) 15–25 (19.5 ± 2.91) long, ‘triangular’ to trapezoid, slightly twisted along longitudinal axis, each with five odontoid processes. Tooth-like structures vary in size, ventrally becoming prominent, overall conferring triangular aspect to crurae. Telamon plates poorly developed, triangular in lateral view, located ventrally to posterior extremity of gubernaculum.

### Females

Based on four intact females, one cephalic and seven caudal extremities. Total length (n = 4) 16.25–21.52 mm (18.3 ± 2.3); maximum width (n = 9) 73–102 (86.0 ± 9.9). Esophagus (n = 5) 270–310 (289 ± 14.71) long and 30–42 (36.7 ± 4.32) wide at base, and (n = 4) 1.3–1.7% (1.6 ± 0.2%) of body length. Nerve-ring (n = 5) 86–97 (91.6 ± 4.33), cervical papillae (n = 3) 150–180, excretory pore (n = 5) 159–220 (190.4 ± 29.11) from cephalic extremity. Uteri paired, prodelphic; sphincter at end of uterine limbs (n = 7) 21.19–35.86 (31.8 ± 3.96) long. Vagina voluminous (n = 8) 702.2–961.42 (846.4 ± 94.94) long, subdivided in vagina uterina (n = 8) 637–889.7 (779.2 ± 93.82) and vagina vera (n = 10) 63.27–71.72 (66.8 ± 2.7) connected by sphincter. Vulval aperture on solid knob-like protuberance; cuticular fold extending ventrally across protuberance from anterior lip of vulva; body width at vulva (n = 12) 45.64–69 (56 ± 7.31). Provagina well developed with a hood-like fold extending ventrally across prominent genital protuberance. Peri-vulval pores disposed bilaterally at level of vulva. Anus in the mid-third of distance between vulva and tail tip; distance vulva-anus (n = 11) 70.1–104 (87.3 ± 10.12); vulva-tail (n = 11) 107.58–146 (131.9 ± 12.77). Tail conical (n = 11) 34.23–50.53 (44.5 ± 4.65) with lateral phasmids near apex.

### Immature stages

***First-stage larvae (DSL)*:** Based on 15 larvae from the lungs of an Eurasian moose. Total length 221.5–373.7 (268.6 ± 40.81). Maximum body width 12.2–29.6 (20.1 ± 5.94). Esophagus 111.6–182.5 (132.2 ± 15.92), 41.2–55.5% (46.4 ± 3.85%) of body length, maximum width at base 6.19–15.7 (10.7 ± 3.51). Body width at esophageal base 10.9–29.6 (19.5 ± 5.95). Nerve-ring 64.5–86.3 (74.1 ± 5.26), excretory pore 70.5–88.9 (78.8 ± 5.33) posterior to cephalic extremity. Genital primordium 145.6–250.6 (202.3 ± 30.69), from anterior end, 54.7–79% (70.7 ± 6.04%) of body length from anterior. Anus-tip of tail spike 34.4–40.4 (37.3 ± 3.03), Anus-insertion of tail spike 19.2–30.3 (26.9 ± 2.95), Tail spike 9.7–12.4 (10.4 ± 0.68) in length with three prominent folds; dorsal spine 2.8–3.5 (3.1 ± 0.24). ***Eggs*:** Spherical to ovoid with delicate, smooth shell (n = 20); 55.2–66.5 (61. 9 ± 3.51) long, 46.2– 63.0 (55.2 ± 6.14) in width.

### Taxonomic summary

#### Type-host

Eurasian moose (*Alces alces*). Other common name: Eurasian elk.

#### Habitat

Adult males and females in terminal bronchioles and alveoli of lungs based on recovery of specimens through dissection of lesions.

#### Type-locality

Original type-locality: Moscow Region, Russia. Additional locality for designated Neotype: Vestby Municipality, Akerhus County, Eastern Norway, Norway (present study). Also known from areas of Sweden, Finland, Poland, and Estonia.

#### Specimens

Neotype male from type host and new designated locality (59°30′N, 10°40′E) collected from lungs of a young male Eurasian moose (V-456) by S. Kutz and others in Norway, USNPC 106337. Voucher specimens collected from the same host, USNPC 106338–106340, and from three other hosts: a young female (V-376), USNPC 106331–106334 (including DSL material); another young female (V-377), USNPC 106335; and an adult female (V-383), USNPC 106336; all from the same locality.

### Differential diagnosis

*Varestrongylus alces* is resurrected based on morphological and molecular character data; and, therefore, this valid taxon must be separated from *V. capreoli.* A neotype is designated herein because name-bearing types were not identified or deposited at the time of the original description [21] and are apparently absent in Russian museum collections (A. Makarikov, pers. comm.). This proposal is consistent with and based on the provisions specified in Article 75, Chapter 16 of the International Code for Zoological Nomenclature [34], with the intent of clarifying the taxonomic status of *V. alces* within the genus.

Consistent with the current generic diagnosis, males of *V. alces* possess a prominent gubernaculum with paired denticulate plates of the crurae disposed slightly lateral, dorsal and distal to the split corpus or legs, and a typical configuration of bursal rays; and females have a well-developed provagina.

Among males, specimens of *V. alces* are readily distinguished by the dimensions and structure of spicules (138.6–163 μm). Spicules of *V. alces* are substantially shorter than those typical of ‘the large spicule group’: *V. alpenae*, *V. capricola*, *V. longispiculatus*, *V. pneumonicus*, *V. qinghaiensis*, *V. sagittatus* and *V. tuvae* (all *>* 300 μm, except *V capricola* whose spicules are approximately 250 μm). Similarly, the gubernaculum (65–83 μm) of *V. alces* is much smaller than that of the aforementioned species (all *>* 100 μm).

Among the Varestrongylinae, *V. alces* is most similar to *V. capreoli* (and *V.* cf. *capreoli*, which is identical to *V. capreoli* but for one character and, therefore, will be mentioned again for comparative matters in this exception) and these two species characterize the small-spicule forms currently known within the genus. Nevertheless, males of *V. alces* differ from those of *V. capreoli* by dimensions of the spicule and gubernaculum as well as several other characters. The conformation of the gubernaculum is the most noticeable difference between *V. alces* and *V. capreoli*; both have a bifurcate corpus, but in the latter, the legs are fused by a transparent membrane that is not observed in the former. In addition, the gubernaculum of *V. alces* does not have a capitulum (head). In contrast, different authors, including the original description [3] and works cited in the most recent revision of the genus [1], regard the presence of a distinctive capitulum of the gubernaculum with two acute ventrally directed projections as typical in *V. capreoli*. Variation, however, may be evident in this attribute as specimens, referred to *V.* cf. *capreoli* in roe deer from the present study lacked a capitulum, suggesting a more extensive series of male nematodes should be evaluated for this character. Among additional characters, spicules of *V. alces* and *V. capreoli* are comparable in length, and morphologically very similar. For both, the alae originate in the first third and extend slightly beyond the distal extremity of each spicule. However, the distal ends of the spicules of *V. alces* are more spatulate than in *V. capreoli*. The denticulate plates of the crurae differ in shape, being slightly twisted and conferring an arched appearance in *V. alces,* with both plates together resembling a horseshoe (Figure 7). In contrast, in *V. capreoli*, the denticulate plates of the crurae are triangular, and more parallel to each other, resembling “Hermes’ wings”. Numbers of denticulate processes in these plates also differ, with *V. alces* having 5 and *V. capreoli* having 3 prominent teeth. The copulatory bursa of *V. alces* is dorsally notched with an indistinct dorsal lobe, whereas the bursa of *V. capreoli* is bi-lobate. A series of subtle differences are also observed in the morphology and disposition of the bursal rays. The dorsal ray in *V. alces* is slightly elongate and bifurcate near its mid-length as opposed to *V. capreoli*, in which the dorsal ray is reduced and rounded, yet still distinguishable. In *V. alces*, the externo-dorsal ray originates independently from the lateral rays, unlike in *V. capreoli*. Ventral rays of both species originate from a common stalk but this is distally split in *V. alces*, whereas it is split near its base in *V. capreoli*.

Measurements for multiple characters overlap between the two species, including some characters that are distinguishable based on morphology (Table 3), but this may be because of the wide range in measurements previously reported for *V. capreoli* [1].

**Table 3:** **Comparative morphometry of males of** ***Varestrongylus alces*** **and** ***V. capreoli***

**Characters**	***V. alces*** ^**a**^	***V. alces*** ^**b**^	***V. capreoli*** ^**c**^	***V.*** **cf.** ***capreoli*** ^**a**^
**Total length**	11.4–14.7 (12.9 ± 2.01)	5–6	5.3–13.5	7.1–8.9 (7.9 ± 0.88)
**Maximum width**	68.5–80 (74.2 ± 4.97)	65	32–68	42–44 (43.5 ± 1.00)
**Esophagus** ^**§**^	250–272 (264.5 ± 8.95)	146	90–146	227–239 (232 ± 5.10)
**Esophagus base width**	32–37 (33.9 ± 2.19)	36	–	20–36 (24.6 ± 6.47)
**Body width at esophagus**	53.8–61.9 (56.3 ± 3.38)	–	–	33–60 (40.4 ± 11.10)
**Nerve-ring** ^**§**^	68–89.7 (81.8 ± 9.04)	–	–	70–81 (76.3 ± 5.60)
**Cervical papillae** ^**§**^	201–207 (203.4 ± 3.19)	–	–	163^*^
**Excretory pore** ^**§**^	208–230.3 (221.8 ± 9.83)	–	–	166–201 (180.5 ± 14.71)
**Spicules**	138.6–163 (152.3 ± 7.31)	150–166	129–160	134–152 (138.3 ± 7.03)
**Gubernaculum**	65–83.13 (76.58 ± 7.06)	–	70–86	70–92 (81.8 ± 8.14)
**Gubernaculum head**	Absent	Absent	Present 8–14	Absent
**Gubernaculum corpus**	38–49 (43.9 ± 4.34)	–	NA	30–38 (32.8 ± 3.77)
**Gubernaculum crura**	24–39.12 (32.6 ± 5.09)	–	NA	32–56 (46.5 ± 10.25)
**Crura denticulate piece**	15–25 (19. 5 ± 2.91)	–	18–30	21–25 (23.2 ± 1.47)
**Body width at bursa**	42–56 (48.3 ± 7.36)	–	–	33–37 (34.5 ± 1.38)
**Bursa width**	125–160 (140 ± 18.03)	–	–	NA
**Bursa length**	75–90 (84.3 ± 5.9)	–	–	NA
**Dorsal ray length**	18–30 (24.5 ± 3.65)	–	NA	6–10 (8.6 ± 1.79)
**Dorsal ray base**	11.4–15 (12.9 ± 1.51)		NA	7.5–12.5 (9.2 ± 2.06)

^a^Present study; ^b^Original description [20]; ^c^Original description [3], plus additional information compiled in [1].

^§^Measurements from anterior end; *Single measurement.

Range of measurements are given followed by mean and standard deviation. Total length in millimeters (mm), and all other measurements are in micrometers (μm).

Among females, the size and shape of the provagina is not always a useful character for discriminating among species of *Varestrongylus*. For instance, the provagina of *V. alces* and *V. capreoli* (and *V.* cf. *capreoli*) is morphologically identical. Similarly, the ranges for maximum body width, and distances between vulva and tip of tail, and anus and tip of tail (tail) for *V. alces* and those for *V. capreoli* largely overlap (Table 4). In contrast when comparing to the roe deer material, identified as *V.* cf. *capreoli*, these measurements, as well as body width at vulva and distance between vulva and anus, are wider or longer in those of *V. alces.* Nevertheless, morphological species identification solely based on female specimens remains challenging.

**Table 4:** **Comparative morphometry of females of** ***Varestrongylus alces*** **and** ***V. capreoli***

**Characters**	***V. alces*** ^**a**^	***V. alces*** ^**b**^	***V. capreoli*** ^**c**^	***V.*** **cf.** ***capreoli*** ^**a**^
**Total length**	16.3–21.5 (18.3 ± 2.3)	11.1–11.5	9.41–15	17.93^*^
**Maximum width**	73–102 (86.0 ± 9.9)	75–95	38–95	48.9–52.2 (50.5 ± 2.31)
**Esophagus** ^**§**^	270–310 (289 ± 14.71)	–	122–290	196–242.9 (225.0 ± 20.21)
**Esophagus base width**	30–42 (36.7 ± 4.32)	–	–	21.9–27.7 (23.8 ± 2.60)
**Body width at esophagus base**	57–67 (61.1 ± 4.56)	–	–	31–40.8 (35.3 ± 5.06)
**Nerve-ring** ^**§**^	86–97 (91.6 ± 4.33)	–	72–90	55.4–65.2 (60.7 ± 4.49)
**Cervical papillae** ^**§**^	150–180 (163.3 ± 15.28)	–	–	185.82^*^
**Excretory pore** ^**§**^	159–220 (190.4 ± 29.11)	–	86–186	171.5–190.8 (183.4 ± 10.37)
**Tail**	34.2–50.5 (44.5 ± 4.65)	–	34–78	31–40.8 (37.2 ± 3.47)
**Vulva-anus**	70.1–104 (87.3 ± 10.1)	–	–	57.1–73.4 (64.3 ± 6.62)
**Vulva-tail**	107.6–146 (131.9 ± 12.77)	122	90–144	91–114.1 (101.6 ± 8.42)
**Width at vulva**	45.6–69 (56 ± 7.31)	–	–	32.2–35.9 (33.4 ± 1.42)
**Vagina**	702.2–961.42 (846.41 ± 94.94)	–	–	467^*^
**Vagina Vera**	63.3–71.7 (66.8 ± 2.70)	–	–	73.4–91.3 (77.4 ± 6.90)
**Vagina Uterina**	637–889.7 (779.2 ± 93.82)	–	–	391.2^*^
**Sphincter**	21.2–35.9 (31.8 ± 3.96)	–	–	24.45^*^
**Eggs Length** ^**†**^	55.2–66.5 (61.9 ± 3.51)	78	56–78	NA
**Eggs Width** ^**†**^	46.2–63.0 (55.6 ± 6.14)	–	37–45	NA

^a^Present study; ^b^Original description [20]; ^c^Original description [3], plus additional information compiled in [1].

^§^Measurements from anterior end; ^†^Eggs collected from lungs of infected Eurasian moose, not inside female uteri; *Single measurements.

Range of measurements are given followed by mean and standard deviation. Total length in millimeters (mm), and all other measurements are in micrometers (μm).

***First-stage larvae (DSL):*** Comparisons among *Varestrongylus* species DSL are provided in Table 5. Comparisons with other members of the Family Protostrongylidae that occur in the same host or which may have overlapping geographic distributions were also included. In general, most of the characteristics overlap in measurement. The wide range for total length of *V. alces* in our study, especially the lower values, may be attributable to the pulmonary origin (vs. feces) and the fact that lungs were frozen before dissection, and collection and preservation of DSL material. Co-infections with *V. alces* and *E. alces* are common; however DSL of *E. alces* and other *Elaphostrongylus* species appear to be consistently longer than those of *V. alces* (Table 5).

**Table 5:** **Comparative morphometrics of first-stage larvae (DSL) of** ***Varestrongylus*** **and of Elaphostrongylinae sympatric with** ***V. alces***

**Characters**	***V. alces*** ^**a,b**^ **(n = 15)**	***V. capreoli*** ^**c**^	***V. sagittatus*** ^**d**^	***V. sagittatus*** ^**e**^	***Varestrongylus*** **sp.** ^**f1**^ **(n = 10)**	***Varestrongylus*** **sp.** ^**f2**^ **(n = 20)**	***V. alpenae*** ^**g**^	***E. alces*** ^**h**^ **(n = 30)**	***E. cervi*** ^**i**^ **(n = 30)**	***E. rangiferi*** ^**j**^ **(n = 15)**
Total length	221.5–373.7	255–341	260–305	268.8–295.7	281–374	348–400	310–380	377–445	392–445	381–490
	(286.6 ± 40.81)	(227–260)	(233–305)	(281 ± 11.9)	(329)	(377)		(417 ± 16)	(420 ± 13)	(426)
Nerve-ring^§^	64.5–86.3	–	–	–	–	78–107	85–93	83–106	106–125	95–130
	(74.1 ± 5.3)					(97)		(90 ± 16)	(114 ± 5)	(110)
Excretory pore^§^	67.5–88.9	–	81–84	77–122.9	71–105	92–107	85–93	104–132	104–121	97–125
	(78.8 ± 5.33)			(96±17.5)	(84.5)	(102)		(112 ± 7)	(111 ± 4)	(109)
Esophagus^§^	111.6–182.5	70–83	115–151	134.4–161.3	88–155	151–180	155–180	173–236	175–206	163–230
	(132.2±15.92)	(120–140)	(124)	(147±15.9)	(128)	(168)		(188 ± 12)	(187 ± 7)	(191)
Esophagus/total length (%)	41.2–55.5	–	–	–	28–46	43–46	47–50	–	–	–
	(46.3±3.85)				(38)	(45)				
Esophagus base width	6.2–15.7	–	–	–	8–15.5	9–15	–	–	–	–
	(10.7 ± 3.51)				(10)	(12)				
Body at esophagus base	10.9–29.6	–	–	–	–	–	–	–	–	–
	(19.5 ± 5.95)									
Max body width	12.2–29.6	10–17	14–17	13.2–16.9	16–23	17–20	15–17	17–21	17–22	17–24
	(20.1± 5.94)	(11–14)	(14)	(15± 1.1)	(19.5)	(18)		(19 ± 1)	(19 ± 1)	(20)
Genital primordium^§^	145.6–250.6	–	179–201	154–249.6	173–224	218–273	195–242	204–289	253–288	245–325
	(202.3 ± 30.69)			(197±25.1)	(206)	(244)		(262 ± 16)	(270 ± 10)	(267)
Genital primordium/total length (%)	69.3–72.9 (70.7±6.04)	–	–	–	62–64	61–68	63–64	–	–	–
					(63)	(65)				
Tail length	28.6–39.4	28–32	25–31	24.64–29.28	31–42	32–41	–	32–49	37–47	32–53
	(36.4 ± 2.95)			(28± 1.63)	(35)	(38)		(42 ± 5)	(43 ± 3)	(44)
Tail spike	9.8–12.4	8	(9–10)	9.2–10.78	8–11	6–12	data not given	data not given	data not given	data not given
	(10.4 ± 0.68)			(9.6± 0.7)	(9)	(9)				
Dorsal spine	2.8–3.5	2	data not given	data not given	1.6–3	data not given	data not given	data not given	data not given	data not given
	(3.1 ± 0.24)				(2)					

^a^Present study – DSL recovered from lung washes and fixed in 70% ethanol and measured at 1000× magnification. The wide range for total length, especially the lower values might be attributable to the pulmonary origin (vs. feces) and fixation method.

^b^only measurements available in the original description [20], were total length, 305–441 μm and maximum width, 12 μm.

^c^Combined sources compiled in [1], origin (lungs/feces) or fixation method not mentioned.

^d^Combined sources compiled in [1], recovered from lungs, fixation method not mentioned.

^e^DSL recovered from feces of red deer from the Vitinya wildlife-breeding station in the west Balkan Mountains, Bulgaria, not fixed and measured after iodine staining [45].

^f^Undescribed *Varestrongylus* species found in caribou, muskoxen and moose across northern North America [14]. DSL recovered from feces of muskoxen from: (f1) Nunavik Region, Quebec, Canada, fixed in 70% ethanol and measured at 1600× magnification, (f2) near Aklavik, Northwest Territories, Canada, heat-relaxed in water and measured at 400× magnification.

^g^ *V. alpenae* DSL extracted from white-tailed deer feces, New York, USA in [45].

^h^DSL recovered from feces of experimentally infected Eurasian elk, material was heat-relaxed in water and measured at 1000× magnification [8].

^i^DSL recovered from feces of experimentally infected red deer, material was heat-relaxed in water and measured at 1000× magnification [8].

^j^DSL recovered from feces of woodland caribou from Newfoundland, Canada. Material was heat-relaxed in water, magnification not mentioned [8].

^§^Measurements from anterior end.

Range of measurements are given followed by mean and standard deviation. Measurements are given in micrometers (μm).

### Molecular identification and phylogenetic comparisons

All ITS-2 sequences generated were deposited in GenBank under accession numbers: KJ452181–96 for *V. alces* of Eurasian moose; KJ452174–80 for *V.* cf. *capreoli* of European roe deer; and KJ439592–98 for *V. sagittatus* isolates in red deer from Bulgaria and are accompanied by vouchers specimens deposited in the USNPC (Table 1). Intra-individual ITS-2 sequence polymorphisms were found for all three *Varestrongylus* species evaluated. The ranges of pairwise similarity among individuals, within species, and between the five *Varestrongylus* species are provided in Table 6.

**Table 6:** **ITS-2 pairwise identity among** ***Varestrongylus*** **species and individuals, including intra-individual variability**

***Varestrongylus*** **species**	***V. alces*** ^*^	***V.*** **cf.** ***capreoli*** ^*^	***Varestrongylus*** **sp.**	***V. alpenae*** **	***V. sagittatus*** ^*^
***Varestrongylus alces***	71.7–99.5 (87.14 ± 6.46)	–	–	–	–
***Varestrongylus*** **cf.** ***capreoli***	64.8–89.6 (78.76 ± 4.73)	78.1–100 (92.85 ± 8.12)	–	–	–
***Varestrongylus*** **sp.**	64.9–87.1 (78.25 ± 4.63)	74.9–84.9 (82.06 ± 1.91)	94.7–100 (97.37 ± 1.73)	–	–
***Varestrongylus alpenae***	57.2–72.8 (63.9 ± 6.5)	64.6–72.5 (63.25 ± 3.65)	72.4–74.7 (74.35 ± 0.92)	100^**^	–
***Varestrongylus sagittatus***	42.1–58.7 (51.92 ± 3.24)	50.3–61.2 (58.33 ± 2.23)	55.4–58.8 (57.47 ± 0.76)	50.8–53.5 (52.35 ± 0.45)	87–100 (92.65 ± 5.24)

*Including clones of the same nematode specimen; **single sequence.

Range, average and standard deviation are given.

The alignment of 53 ITS-2 sequences of 12 Protostrongylidae taxa resulted in a dataset of 210 characters. The strict consensus of the three most-parsimonious trees had a length of 271 steps, a consistency index of 0.73, and yielded five monophyletic groups of *Varestrongylus*, each matching pre-determined taxa at representing discrete species. The MP analysis of ITS-2 sequences (Figure 1) strongly support the reciprocal monophyly of *V. alces* isolates (91% bootstrap support), and hence independence from *V.* cf. *capreoli*, and by extrapolation, from *V. capreoli* (*sensu* Stroh and Schmid [3])*.* Clonal sequences of *V.* cf. *capreoli* (92%) and *V. sagittatus* (99%) also formed strongly supported monophyletic clades, confirming their validity as independent taxa. Moreover, the DSL-derived ITS-2 sequences for an undescribed *Varestrongylus* strongly supported recognition of a previously unknown species and confirmed its placement within the genus (97%) [15,17].

*Varestrongylus alces* formed a well-supported clade with this undescribed Nearctic species and *V.* cf. *capreoli* (80%), but relationships among these three species were equivocal. A sister relationship of *V. alpenae* to the clade formed by *V. alces*, *V.* cf. *capreoli* and the undescribed North American species was also well supported (81%). *Varestrongylus sagittatus*, a parasite of Cervinae, is sister for a clade formed by the four *Varestrongylus* species parasitic in Odocoileinae cervids (Figure 1). Sequences from species within the subfamilies Elaphostrongylinae (99%), Muelleriinae (84%) and Protostrongylinae (99%) also formed well supported clades.

### Pathology

#### Gross pathology

**Figure 9:**
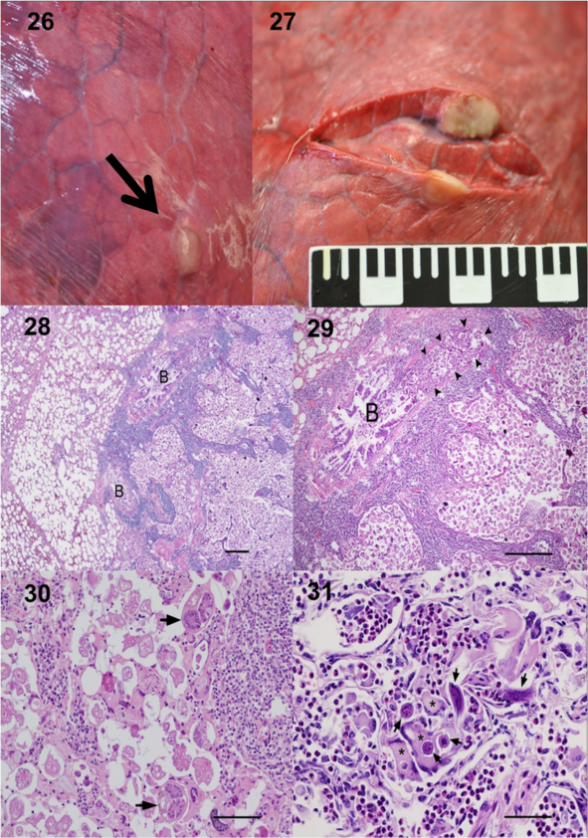
**Gross and histopathological changes in lungs of Eurasian moose infected with** ***Varestrongylus alces.*** 26, 27. Gross lesion seen from lung surface during gross examination (arrow), typical of varestrongylosis (26), and sectioned lesion (≈1.5 cm) (27). 28–31. Histological sections (H&E). 28. Part of the nodule is seen to the right, consisting mainly of large amounts of eggs, larvae and inflammatory cells, whereas normal, slightly emphysematous tissue is seen to the left. Scale-bar: 500 μm. 29. A close up of 28 showing to the left a large bronchiole (B) with epithelial hyperplasia and peri-bronchiolar lymphocytic inflammation that has large amounts of larvae in the lumen (area surrounded by arrowheads). To the right numerous eggs and larvae are filling up the alveolar space with rupture of alveolar septa and infiltration of inflammatory cells, mainly interstitially. Scale-bar: 500 μm. 30. Cross sections of adult nematodes (arrows) in the alveolar lumen surrounded by large amounts of eggs and some larvae with scattered multinucleated giant cells. Scale-bar: 100 μm. 31. First-stage larvae (arrows) partly engulfed and surrounded by giant cells (*), some macrophages and numerous eosinophilic granulocytes. Scale-bar: 50 μm.

Grossly, lesions in Eurasian moose lungs were well defined, tan to pale and firm nodular lesions that ranged in size from a few millimetres to 2–3 cm in diameter. These were mostly seen subpleurally, but could also be found deeper in the lung tissue (Figure 9). Most lesions were found in the caudo-dorsal region of the diaphragmatic lobes. Lesions were clearly demarcated against adjacent normal lung tissue.

#### Histopathological findings

Histological examination revealed acute to sub-acute focal verminous pneumonia restricted to one or a few neighboring lobules (Figure 9). Within the affected lobules, large numbers of eggs and larvae, some of them degenerated and mineralized, were filling up the alveolar lumen with rupture of alveolar septa. Numerous larvae were also seen in the lumen of some of the surrounding large bronchioles (Figure 9). Scattered cross sections of adult nematodes were found in the alveoli (Figure 9). Reactive changes included infiltration of variable amounts of multinucleated giant cells, macrophages, eosinophilic granulocytes and lymphocytes (Figure 9). Marked interstitial infiltrations of inflammatory cells, dominated by lymphocytes and macrophages, were evident around bronchioles and vessels and in the remaining alveolar septa surrounding islands of ruptured alveoli filled with eggs and larvae. Bronchioles with larvae in the lumen had mild hyperplasia of the epithelium and inflammation of the wall. The overlying pleura and the interlobular septa showed variable degree of fibrosis and infiltration of inflammatory cells dominated by lymphocytes.

In adjacent tissue, a few scattered eggs and larvae in the alveolar lumen with little reactive changes (microgranulomas) were seen, as typically found in *E. alces* infection [28].

## Discussion

### Species identity

*Varestrongylus alces* is a valid species based on combined morphological and molecular evidence, corroborating the findings of the original species description [21] and, therefore, should be separated from *V. capreoli*, as postulated in the last revision of the genus [1]. Given that the types were either never deposited in a Russian museum repository (there is no indication in the original description), or have been subsequently lost, we propose designation of neotype for *V. alces*. Such a proposal serves to clearly validate the species, distinguishing this taxon among its congeners, and establishes stability in the current nomenclature for this group of nematodes.

As for many taxa within Protostrongylidae, and especially within the genus *Varestrongylus*, the taxonomic history of *V. alces* has been confusing [1]. Despite the widely accepted synonymy with *V. capreoli*, a few authors have continued to use *V. alces* as a valid taxon, however, without emphasizing its dubious taxonomic status and not focusing on aspects of its life history. Others did not follow the proposed revision at the generic level made by Boev [20], in which *Capreocaulus* Schulz & Kadenazy, 1948 and *Bicaulus* Schulz & Boev, 1940, were regarded as junior synonyms of *Varestrongylus.* Adding to the confusion, studies that disregarded the species-level synonymy have placed both species in two separate genera: *Capreocaulus* for *V. capreoli* (as *Capreocaulus capreoli* (Stroh & Schmid [3]) Schulz & Kadenazy, 1948)) [22,25,26] and *Bicaulus* Schulz & Boev, 1940 for *V. alces* (as *Bicaulus alces* (Demidova & Naumitscheva, 1953) Boev, 1957 or *B. alcis* (sic)) [27,35]. Such inconsistencies reinforced our need to resolve the taxonomy and the possible synonymy or independence of *V. alces* and *V. capreoli* [1], given recognition of an unknown taxon in related hosts from North America.

### Molecular findings

Sequences at the ITS-2 locus of *V. alces* formed a strongly supported monophyletic group, and were distinct from those of *V.* cf. *capreoli*, and all *Varestrongylus* species from which sequences were available. According to the most parsimonious tree, *V. alces* is the sister taxon of *V.* cf. *capreoli*. These two species form a well-supported clade with the undescribed *Varestrongylus* from the Nearctic, and are more distantly related to *V. alpenae* and *V. sagittatus*. The multiple sequences of *V.* cf. *capreoli*, *V. sagittatus* (clones from this study), and the undescribed Nearctic species (from [15]) also formed strong monophyletic clades, supporting species identity. In the only previous attempt to apply molecular or genetic data in comparisons of *Varestrongylus* isolates from *Alces* and *Capreolus* hosts [35], protein band patterns and their protein isoeletric points were used to distinguish protostrongylid larvae from different host sources. Isolates attributable to *V. alces* were closely related, but not identical, to those of *V. capreoli* (referred as *C. capreoli*) when contrasted to larval isolates from muskoxen and elaphostrongylines in Eurasian moose and reindeer, consistent with our findings in the present study.

The intra-individual ITS-2 variability we found for *V. alces*, *V.* cf*. capreoli* and *V. sagittatus* is not surprising as it is a multi-copy gene [36]. In fact, variability at this region has been demonstrated in members of the Family Protostrongylidae and the undescribed Nearctic *Varestrongylus* species [15], and the intra-species diversity is expected to increase with the number of individual worms and clones sequenced. Intra-individual variability in multi-copy genes, such as the ITS-2 region, appears to be common in parasitic nematode species and other organisms, and it may indicate incomplete rDNA repeat homogenization within these species [36]. Similar patterns have been reported for *Nematodirus battus* Crofton & Thomas 1951 [37], as well as for various other gastrointestinal strongylid species infecting domestic and wild mammals [38-43]. Conversely, all clones from the two *V. sagittatus* specimens showed minimal variability within and between specimens.

### Pathology and significance

Gross and histopathological pathological lesions found in *V. alces*-infected moose in the present study resembled those described for several other congeneric species, such as *V. capreoli* [3,44], *V. pneumonicus* [4], *V. alpenae* [45], and *V. sagittatus* [46], and previous reports for *V. alces* [5,23,24]. Since adult *Varestrongylus* are often found in small bronchioles, infection is generally associated with focal or multi-focal pneumonia, most often in the diaphragmatic lobes [1,45], as opposed to the diffuse interstitial pneumonia typical of the non-pulmonary protostrongylids (i.e., elaphostrongylines), where larvae and eggs are disseminated into the lungs via blood stream [47]. Perhaps, the similar pulmonary pathology caused by *V. capreoli* in European roe deer ([1,3,44]; S. Kutz, unpubl obs.]) may have influenced Boev and other Russian parasitologists in making *V. alces* a junior synonym of the former, together with the previously mentioned reasons.

*Varestrongylus alces* is a common parasite in moose in Norway, with reported prevalence ranging from 8% to 26% [23,24]. The infection occurs as an incidental autopsy finding in moose dying from various causes and has never been associated with disease in moose in this country. It could, however, be speculated that heavy *V. alces* infection may predispose the lungs to secondary bacterial infections. This could also be the case if this parasite occurs in combination with *Dictyocaulus* and *E. alces*, as observed in at least two animals in the present study. Co-infection of *V. alces* and *E. alces* appears to be relatively common in moose both in Norway [23,24], and other European countries [22,25]. Parasitism by multiple species of lungworms and/or extra-pulmonary protostrongylids may produce cumulative or synergistic deleterious effects, as suggested in cases of co-infection in different host-parasite systems [16,47-49].

### Biogeography – past and present

*Varestrongylus alces* appears to be geographically restricted to the Palaearctic. To date, the parasite has been recognized in *A. alces* from at least six European countries: Poland [22], Norway ([23,24]; present study]), Sweden [25], Finland (cited in [27]), Estonia [26], and areas of western Russia [5,21]. *Varestrongylus alces* has not been reported from subspecies of *A. americanus* in eastern Russia, although the search effort for the parasite is not known. In North America, despite reasonably extensive fecal surveys of North American moose in northern Canada and Alaska, it has not been found (revised in [16]; G. Verocai, unpubl. data). In the absence of extensive geographic and host sampling, we can look to host and parasite phylogeny and host historical biogeography to develop and explore hypotheses about the geographic distribution and host associations for *V. alces*.

Recent genetic evidence supports that *Alces* comprises two extant species: *A. alces,* referred as Siberian moose or elk as per the International Union for Conservation of Nature (IUCN) [50], in Central Russia to Europe, and *Alces americanus* (Clinton 1822)*,* referred as moose, in eastern Asia and North America [9,51-53] (or two major genetic clades, but only different subspecies [51])*.* The contemporary distribution of *Alces* is a result of complex historical patterns of geographic expansion and retraction, and isolation. *Alces* survived the glaciations of the Pleistocene in multiple refugia south of the ice-sheets, as supported by fossil records within Europe and Asia [54-56]. Throughout the Pleistocene and early Holocene, the distribution of *A. alces* in Europe was considerably broader, comprising many countries of western and central Europe, as per fossil and sub-fossil findings. After recession of the continental ice-sheet, *A. alces* recolonized much of the previously glaciated regions of Eastern Europe, Fennoscandia and Russia, and concomitantly went extinct in areas of Western and Central Europe [57,58]. In more recent times, *A. alces* was nearly extirpated in Europe and only recolonized its current range after the World War II. This population bottleneck, followed by recent geographic expansion, resulted in the low genetic diversity seen among extant populations [56]. Nonetheless, *V. alces* seems to have persisted in regions represented by the different genetic clades reported in this study, and potentially recolonized suitable areas from Fennoscandia and eastern Europe, extending eastwards to the Central Russian Federation, Kazakhstan, Northern China and Mongolia [53] along with its definitive host.

Siberian moose in Eastern Asia are conspecific to the North American moose subspecies. Historical processes that shaped this divergence and lead to speciation within *Alces* may explain the absence of *V. alces* in *A. americanus* from Eastern Asia and, consequently, North America, to where *Alces* expanded and colonized only during the Late Pleistocene [9,11]. If the association with *Alces* is evolutionarily deep, *V. alces* or its ancestor may have been lost in *A. americanus* populations due to ecological factors. Alternatively, in case of a more shallow association, *V. alces* or its ancestor could have host-switched, and established in *A. alces* after isolation, and allopatric speciation when this host was in sympatry with other cervids associated with *Varestrongylus* species**.**

Current literature and the knowledge of the historical biogeography of *Alces* and other ungulates may support an exclusive contemporary association of *V. alces* to the Eurasian moose; potentially suggesting a deep historical association with this host. Historically, during the Pleistocene, *Alces* was sympatric with other cervids including *Cervus elaphus*, *Capreolus capreolus,* and *Rangifer tarandus* [55,59] in several temperate refugia within Eurasia. Coincidentally, all these cervids are hosts for other *Varestrongylus* species in Eurasia or North America. Additionally, this historical host sympatry indicates the long-term co-existence of different *Varestrongylus/*Cervidae assemblages, which support an early diversification within the parasite genus, perhaps congruent to ungulate diversification. Alternatively, this extensive sympatry and diversity of the mammalian megafauna may also have facilitated the occurrence of host-switching among ungulate hosts. Glacial cycles during the Pleistocene caused the extinction of multiple elements of this mammalian community and promoted isolation in different refugia, also altering patterns of sympatry of cervid hosts of *Varestrongylus* species, as mentioned above. As proposed by Hoberg et al. [60], increased allopatry and host extinction events could have: (i) resulted in lowered diversity in certain parasite groups, as in the monospecific genus *Umingmakstrongylus* Hoberg, Polley, Gunn & Nishi, 1995, or (ii) constituted a determinant of post-glacial isolation and allopatric speciation of certain parasites, which may be the case of the diverse genus *Varestrongylus*.

This apparent absence from North American moose is perhaps not surprising, as there is no overlap between Eurasian and Nearctic protostrongylid fauna, with the exception of cases where there have been anthropogenic introductions [1,8,11,16,31]. Nevertheless, North American moose are incidental hosts for many protostrongylids: *Orthostrongylus macrotis* (Dikmans 1931) Dougherty and Goble [19,61], *Parelaphostrongylus tenuis* (Dougherty [18]) [62], *Parelaphostrongylus andersoni* Prestwood 1971, the introduced Eurasian protostrongylid *E. rangiferi* (Mitskevitch 1960) [63], and the undescribed Nearctic *Varestrongylus* species [15].

Whether *V. alces* is exclusively associated with *Alces* or if other contemporary sympatric cervids may serve as suitable hosts is still unclear. Herein, we consider previous reports of *V. capreoli* in Eurasian moose suspect, and more likely to be *V. alces,* as specimens were not confirmed by morphological or molecular identification; vouchers do not exist in museum collections from these surveys. Future studies should use combined morphological and molecular approaches to unequivocally diagnose *V. alces* and *V. capreoli,* and further assess their host specificity, especially in areas of sympatry. Further it is critical that any field collections be accompanied by deposition of specimens which make it possible to apply integrated approaches to assessments of parasite diversity [64].

There is a relatively close genetic association of *V. alces* to the undescribed, multi-host, *Varestrongylus* species whose putative primary host is the caribou and appears to be geographically restricted to the Nearctic [15,49]. This may suggest its potential infectivity to other hosts, in particular reindeer. Recently, pulmonary lesions compatible with those caused by *Varestrongylus* species were found in reindeer in Finland (Antti Oksanen, pers. comm.). In regions of Fennoscandia and Russia, the geographic range of the Eurasian moose overlaps with reindeer and it is conceivable that *V. alces* can persist in both hosts. Alternatively, the lesions may be associated with infection by the newly described *Varestrongylus* sp. from North American caribou despite no gross pulmonary lesions have been ever observed caribou or muskoxen examined for this lungworm species [15,16], or could be caused by yet another cryptic species of *Varestrongylus* circulating in Eurasian reindeer.

### *Varestrongylus* cf. *capreoli – V. capreoli* as a species complex?

In our study, the male specimens recovered from lungs of roe deer were largely consistent with *V. capreoli* (*sensu* Stroh & Schmid [3]) but differed based on one structural character, the absence of a capitulum/head of the gubernaculum. Such remarkable intra-specific morphological variations have not previously been described for *Varestrongylus* species or other protostrongylids [1]. This morphological difference was consistent across specimens and led us to identify these as *V.* cf. *capreoli.* In *V. capreoli*, the head of gubernaculum in males is considered not only as a diagnostic feature, but as an autapomorphy of this species, that is, a unique feature not shared within *Varestrongylus,* potentially due to an independent evolutionary trajectory (speciation) and, therefore, has been considered of phylogenetic relevance [2]. Notably, we did not have access to any archival material of *V. capreoli*, and could not verify the original description of the capitulum. Morphologically, females of *V.* cf. *capreoli* are virtually indistinguishable from either *V. capreoli*, and *V. alces.* The wide range of measurements reported for *V. capreoli* [1] could be hiding either a species complex or simply represent intra-specific variability (i.e. the existence of morphotypes or lineages in males). However, as in the case of *V. alces*, morphologically similar species could have been equivocally identified as, or arbitrarily synonymized with, *V. capreoli*. Additionally, supporting the potential existence of a species complex within *V. capreoli* is its apparent broad host range, as it has been reported in sympatric caprine hosts: the mouflon (*Ovis aries*) in Czech Republic and domestic goats in the Swiss Alps [65,66] cited in [1]. These reports may be equivocal and are yet to be confirmed. Conversely, the recent finding of *Varestrongylus* species that infects caribou, muskoxen (caprine) and rarely moose in North America [15], could support a potentially wide host range for *V. capreoli*.

To address this emerging question, *V. capreoli*-like material of cervid and caprine hosts from the type locality (Bavaria, Germany) and other Eurasian regions should be assessed by combined morphological and molecular approaches. A first step would be to retrieve ITS-2 sequences of male *V. capreoli* that possess the capitulum of the gubernaculum for comparative analysis, and later evaluate multiple genetic markers. In this way, it would be possible to determine if these different morphological features are only intra-specific variation or if there is a cryptic *Varestrongylus* species in roe deer from Norway and other areas of Fennoscandia, reflecting perhaps a more recent event of geographic isolation of parasite populations and speciation within the same host. In recent history, after periods of population fluctuations, roe deer in Fennoscandia were reduced to less than 100 individuals concentrated in the southernmost part of the Scandinavian Peninsula (Southern Scandia, Sweden) [67,68]. From the 1850’s onwards roe population expanded, and now occupies most of Norway, Sweden and Finland [68]. This recent and drastic host population bottleneck could have resulted in the genetic drift of this heritable polymorphic gubernaculum in males of *V. capreoli* in the region, or alternatively, this polymorphism may be attributed to natural selection.

### Final remarks

Further comprehensive investigation targeting *Varestrongylus* hosts in Eurasia and North America (i.e. cervids and caprines) in conjunction with a systematic reassessment of the taxonomic status of dubious taxa through integrated classical and molecular methods in parasitology may reveal an even richer hidden biodiversity within *Varestrongylus.* Consequently, such investigation would give us a better understanding on the historical biogeography and relationships among the species within the genus, their associations with different ungulate hosts, and, ultimately, provide valuable insights on the historical biogeography of ungulate species.

The use of appropriate molecular markers for species-level identification is a powerful tool for discriminating valid species among cryptic species complexes [36,69,70]. In this study, molecular analysis, combined with classical methods, assisted us in re-examining the taxonomic status of a valid species erroneously reduced as a junior-synonym. In addition to their irrefutable similar morphology, other factors that led to this synonymy were the incomplete description and the absence of species types, or vouchers, deposited in a museum collection, hence the importance of specimen deposition [71]. Molecular information is relatively scarce for members of the genus *Varestrongylus*, and there is a need to produce new data for species, and ideally, this should be done concurrently from specimens with matching morphologic identification, (i.e. adults). After that, larvae confirmed as belonging to a given species could be used to assess its geographic and host ranges, and may provide relevant material for studies on the species historical biogeography and phylogeography, in conjunction with the history of host-parasite assemblages.

## Conclusions

*Varestrongylus alces* is a valid species, and should be considered separate from *V. capreoli*. Phylogenetic relationships among *Varestrongylus* species from Eurasia and North America are complex and consistent with faunal assembly involving recurrent events of geographic expansion and host switching and subsequent speciation.
